# Identification of SUMOylation-related biomarkers in papillary thyroid carcinoma

**DOI:** 10.1186/s12935-024-03323-3

**Published:** 2024-04-27

**Authors:** Xiang Li, Zigang Ding, Yun Tong

**Affiliations:** 1 Department of General Surgery, The Affiliated Hospital of Jiujiang University, Jiujiang, China; 2 Department of Pain, The Affiliated Hospital of Jiujiang University, No. 57 East Xunyang Road, Jiujiang, 332000 Jiangxi China

**Keywords:** Papillary thyroid carcinoma, SUMOylation, Biomarker, Differentially expressed genes

## Abstract

**Background:**

Small ubiquitin-like modifier (SUMO) modification is increasingly recognized as critical in tumorigenesis and progression. This study identifies biomarkers linked to SUMOylation in papillary thyroid carcinoma (PTC), aiming to advance therapeutic and prognostic strategies.

**Methods:**

Employing PTC datasets and SUMO related genes (SRGs), we utilized univariate Cox regression for prognosis-related SRGs, conducted differential expression analyses, and integrated findings to pinpoint candidate genes. These genes underwent further validation through survival, gene set enrichment, immune infiltration, and drug sensitivity analyses, including external validation via quantitative RT-qPCR. In our final step, we conducted immunohistochemical staining on tumor samples from PTC patients at our center and integrated this with their clinical data to validate BMP8A’s effectiveness in predicting recurrence in PTC.

**Results:**

Three biomarkers—BMP8A, RGS8, and SERPIND1—emerged as significant. Gene Set Enrichment Analysis (GSEA) showed their involvement in immune-related pathways, with differential immune infiltration patterns and drug response correlations observed, underscoring their potential for targeted therapy. Lastly, we validated the efficacy of BMP8A in predicting the recurrence of PTC in patients using clinical and pathological data from our center.

**Conclusion:**

The study identifies BMP8A, RGS8, and SERPIND1 as key biomarkers associated with SUMOylation in PTC. Their linkage to immune response and drug sensitivity highlights their importance as targets for therapeutic intervention and prognosis in PTC research.

**Supplementary Information:**

The online version contains supplementary material available at 10.1186/s12935-024-03323-3.

## Background

Within the global context, the incidence of thyroid cancer has been incrementally rising, establishing it as the most prevalent malignancy in the head and neck region [1, 2]. Papillary thyroid carcinoma (PTC), constituting 80% of all thyroid cancer cases [3], is noted for its lower malignancy and generally favorable prognosis. However, PTC frequently undergoes lymph node metastasis in its early stages, posing significant treatment challenges [4, 5]. The primary treatment regimen includes surgery, supplemented by postoperative thyroid-stimulating hormone (TSH) suppression therapy and radioactive iodine treatment, which, while effective, does not prevent recurrence or mortality in all cases [6–8]. Consequently, identifying new biomarkers and understanding their mechanisms in PTC are crucial for improving early diagnosis and treatment strategies.

Small ubiquitin-like modifier modification (SUMOylation) is a pivotal post-translational modification that involves the conjugation of substrate proteins to small ubiquitin-like modifier (SUMO) proteins via a cascade of enzymatic reactions. This modification significantly influences the activity and function of substrate proteins, impacting various cellular functions [9]. SUMOylation is instrumental in regulating a myriad of biological processes such as cell proliferation, apoptosis, carcinogenesis, and DNA damage repair [10, 11]. Notably, it is intricately linked with the oncogenesis and progression of diverse cancers [12]. Studies have revealed that disruptions in SUMOylation lead to pronounced mitotic anomalies in mice [13] and increased genomic instability due to perturbations in the SUMO signaling pathway [14]. These aspects are critical in tumorigenesis. Moreover, SUMOylation is implicated in the metabolic reprogramming and growth of tumor cells; deficiencies in this pathway are associated with diminished mitochondrial oxidative phosphorylation, enhanced glycolysis, and increased lactate production, all of which contribute to tumor proliferation [15]. Recent investigations have also illuminated a significant association between SUMOylation and the anti-tumor immune response, particularly involving macrophages [16, 17]. Thus, SUMOylation emerges as a potential therapeutic target, offering new perspectives for cancer diagnosis, treatment, and prognosis. Despite its recognized importance, the specific role of SUMOylation in papillary thyroid carcinoma (PTC) remains underexplored and warrants further investigation.

Consequently, the principal objective of our current research is to identify and characterize potential SUMOylation-related biomarkers in papillary thyroid carcinoma (PTC). This involves a comprehensive analysis of the biomarkers’ functions, their implications on the immune microenvironment, as well as an examination of their mutations and drug sensitivity. By delving into these aspects, our study aims to contribute novel theoretical insights into the diagnosis and therapeutic strategies for PTC, potentially leading to more effective and personalized treatment approaches.

## Materials and methods

### Data source

The gene expression matrix and survival data of the The Cancer Genome Atlas (TCGA)-thyroid cancer (THCA) dataset were retrieved from the UCSC Xena platform (https://xenabrowser.net/datapages/), and then the tumor data containing only PTC was obtained by filtering the tumor data from the THCA data. A total of 560 samples were obtained from the TCGA-PTC gene expression matrix, including 502 PTC tumor samples and 58 control samples. The GSE60542 dataset was sourced from Gene Expression Omnibus (GEO) database (http://www.ncbi.nlm.nih.gov/geo/), including 33 PTC tumor tissue samples and 30 normal thyroid tissue samples. In addition, totally 209 SUMOylation-related genes (SRGs) were obtained by searching “SUMOylation” in the Molecular Signatures Database (MsigDB, http://www.broadinstitute.org/gsea/msigdb/index.jsp).

### Differential expression analysis

In TCGA-PTC, differential expression analysis was completed to screen differentially expressed genes (DEGs) between the PTC tumor tissue and control tissue samples by the ‘DESeq2’ R package (version 1.38.0) [18], with the threshold values of |log2FC| > 0.5 and P.adj < 0.05. The volcano plot and heatmap of DEGs were drawn using the ‘ggplot2’ R package (version 3.4.1) [19].

### Identification of SUMOylation related-subtypes

To obtain prognosis-related SRGs, SRGs expression was extracted in TCGA-PTC, and then prognosis-related SRGs were screened by univariate Cox regression analysis (*P* < 0.05). Consensus clustering was an extensively utilized unsupervised method for classifying subtype. In this study, PTC tumor samples in TCGA-PTC were clustered by consensus clustering analysis based on prognosis-related SRGs using the ‘ConsensusClusterPlus’ R package (Version1.62.0) [20] to identify SUMOylation-related subtypes.

The dimensionality reduction analysis was finished using t-distributed stochastic neighbor embedding (t-SNE) to plot the point map of the different subtypes. Subsequently, to explore survival differences between the different subtypes, survival analysis of different subtypes was completed in TCGA-PTC. Expression heatmap of prognosis-related SRGs was drawn for different subtypes based on different clinical characteristics (age, gender, race and survival status) to demonstrate different clinical information and gene expression. Differential expression analysis was performed between the two subtypes with the most significant survival differences using the ‘DESeq2’ R package, and DEGs were screened by setting |log2FC| > 1.0 and P.adj < 0.05. To better visualize the differences in gene expression between the two subtypes, volcano plot was drawn using the ‘ggplot2’ R package.

### Identification of differential genes associated with SRGs

The overlap analysis of DEGs between the PTC tumor tissue and control tissue samples and DEGs between two subtypes was performed, and the intersected genes were regarded as differential genes associated with SRGs. Subsequently, Gene Ontology (GO) and Kyoto Encyclopedia of Genes and Genomes (KEGG) pathway enrichment analyses were conducted on differential genes associated with SRGs by using ‘clusterProfiler’ R package (Version4.7.1.3) [21].

### Identification of biomarkers

Differential genes associated with SRGs were screened by utilizing two machine learning algorithms. The feature genes were screened by incorporating the differential genes associated with SRGs into machine learning algorithms, including least absolute shrinkage and selection operator (LASSO) and support vector machine recursive feature elimination (SVM-RFE). The LASSO and SVM-RFE methods were performed using the ‘glmnet’ (Version4.1.4) [22] and ‘caret’ (Version6.0.93) R packages, respectively. Then, the feature genes identified from the machine learning algorithms were overlapped using ‘ggvenn’ R package (Version0.1.9) to obtain candidate genes.

Subsequently, the receiver operating characteristic (ROC) curves of the candidate genes were plotted in the TCGA-PTC and GSE60542 datasets using the ‘pROC’ R package (version1.18.0) [23], respectively. Candidate genes with area under the curve (AUC) of ROC greater than 0.8 were utilized as candidate biomarkers. Next, the different biomarkers were grouped according to their optimal cutoff values for expression in TCGA-PTC, and then the Kaplan-Meier (K-M) survival curves of different groups of PTC patients were plotted. Candidate biomarkers with *P* < 0.05 were screened as the biomarkers for subsequent analysis.

### Gene set enrichment analysis (GSEA) of biomarkers

To further explore the potential biological functions of the biomarkers, GSEA of the biomarkers was performed. First, the samples in TCGA-PTC were categorized into high and low expression groups based on the biomarker expression. Next, differential expression analysis between high and low expression groups was accomplished using ‘DESeq2’ R package, and log2FC values was calculated. Subsequently, log2FC were sorted from large to small. Then GSEA was conducted with ‘clusterProfiler’ R package, and the reference gene set was the HALLMARK gene set in the MSigDB database. Thresholds were set at |NES| > 1 and *P* < 0.05.

### Immune infiltration analysis of biomarkers

To further explore the relationship between biomarkers and the immune microenvironment, the relative abundance of immune-infiltrating cells was assessed for all samples in TCGA-PTC using CIBERSORT algorithm [24]. Then, Wilcoxon-test was utilized to evaluate the difference in immune cell infiltration between different samples (*P* < 0.05), and the immune scores of immune cells in different samples were shown by drawing box plots. Meanwhile, the correlation analysis among immune infiltrating cells was completed using the ‘corrplot’ R package. Finally, the Spearman correlation analysis was finished between immune cells and biomarkers.

### Drug sensitivity analysis

Calculate the 50% inhibitory concentration (IC50) values of 138 commonly used chemotherapy and targeted therapy drugs from the Genomics of Drug Sensitivity in Cancer (GDSC) database for all TCGA-PTC patients using the “prophytic” R package (version 0.5) [25]. The Spearman correlation analysis was performed to screen drugs associated with biomarkers (|cor| > 0.5). Meanwhile, the samples in TCGA-PTC were categorized into high and low groups according to the expression of different biomarkers, and the IC50 differences of the drugs between different biomarker high and low expression groups were compared by Wilcoxon-test.

### Validation of biomarker expression

To assess the robustness of biomarker expression across diverse datasets, we initially compared the expression levels of biomarkers between PTC and control samples in both the TCGA-PTC and GSE60542 datasets.

Subsequently, validation was performed using clinical specimens obtained from 20 patients who underwent surgical procedures at our institution. This included both PTC tissue and adjacent normal tissue, which were promptly stored at -80 °C.

Extract total RNA from the collected tissue using TRIzol reagent, followed by reverse transcription using PrimeScript-RT-Master Mix. Analyze the expression of target genes using the SYBR Premium Ex Taq II PCR detection system, with GAPDH serving as an internal control reference. Calculate the normalized expression level using the 2^-ΔΔCT method to determine the relative fold change. Refer to Table 1 for the primer sequences.

**Table 1:** Consists of a collection of primer sequences utilized in this study

Gene	Sequences
BMP8A F	GGGGCAGAAATACGAGAA
BMP8A R	CAGAAAGGTGGAGTTGGAG
RGS8 F	GGGAGGCAAGAAGTAA
RGS8 R	TTCAGTATCTGGCATCAA
SERPIND1 F	TCAAAGCCACAGGGAA
SERPIND1 R	CTGAAGCCGAACCATT
GAPDH F	CAGTGGCAAAGTGGAGATTGTTG
GAPDH R	TCGCTCCTGGAAGATGGTGAT

### Validate the relationship between BMP8A expression and recurrence risk stratification based on patient clinical data

A retrospective analysis was conducted on 120 patients diagnosed with PTC at the Affiliated Hospital of Jiujiang University between September 2022 and August 2023. The inclusion criteria for the study were as follows: patients who had undergone surgery at the hospital, confirmed PTC through postoperative pathology, and had complete clinical data. Patients with other malignant tumors, postoperative pathology confirming different types of thyroid cancer, missed diagnosis, or incomplete clinical information were excluded. Subsequently, clinical data were collected, and immunohistochemical staining of tumor tissues was performed on these 120 patients.

In the immunohistochemical process, all tissue specimens were sectioned into continuous slices of 4 μm. The sections underwent a series of procedures including dewaxing, rehydration, antigen retrieval, and blocking with goat serum. Subsequently, they were incubated overnight at 4 °C with a mouse anti-human BMP8A polyclonal antibody. The sections were then washed with sterile Phosphate Buffered Saline (PBS) and incubated with the secondary antibody at room temperature for one hour. Following the addition of the substrate and counterstaining with hematoxylin, the sections were observed under a microscope. The immunohistochemical results were independently recorded and reviewed by two experienced pathologists. The presence of brown or brownish-yellow granules in the cytoplasm or on the cell membrane was marked as positive. Cells without staining were marked as negative.

The pathological staging and recurrence risk stratification of PTC patients were based on the latest AJCC guidelines for TNM staging and the ATA guidelines for recurrence risk stratification. Accordingly, we categorized the patients into low-risk and intermediate-high-risk groups.

### Statistical analysis

The analysis was conducted using R software (version 3.6.1; available at https://www.r-project.org/). The relationship between categorical variables was verified using the Chi-square test. Variables deemed potentially significant in univariate analysis were included in multivariate analysis, and their significance was validated using logistic regression analysis. A *p*-value of less than 0.05 was considered statistically significant.

## Results

### DEGs1 and prognosis-related SRGs were obtained

**Fig. 1:**
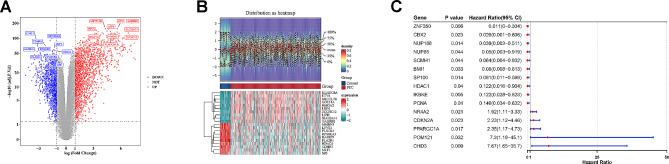
Identification of DEGs and prognosis-related SRGs (**A**) Volcano map of DEGs1 (Differentially expressed genes between PTC and control tissues); (**B**) Heat map of DEGs1 (Differentially expressed genes between PTC and control tissues); (**C**) Forest map of prognosis-related SRGs in patients with PTC

A total of 2,984 DEGs1 (PTC vs. control) were identified in TCGA-PTC, including 1,783 up-regulated genes and 1,201 down-regulated genes, and the top 10 up-regulated and down-regulated genes were shown by volcano maps and heat maps (Fig. 1A-B). By univariate Cox regression analysis, 22 prognosis-related SRGs were screened with the threshold at *P* < 0.05 (Supplementary Table S1), and the genes with the top 15 h values were displayed (Fig. 1C).

### SUMOylation related-subtypes were obtained through consensus clustering analysis

**Fig. 2:**
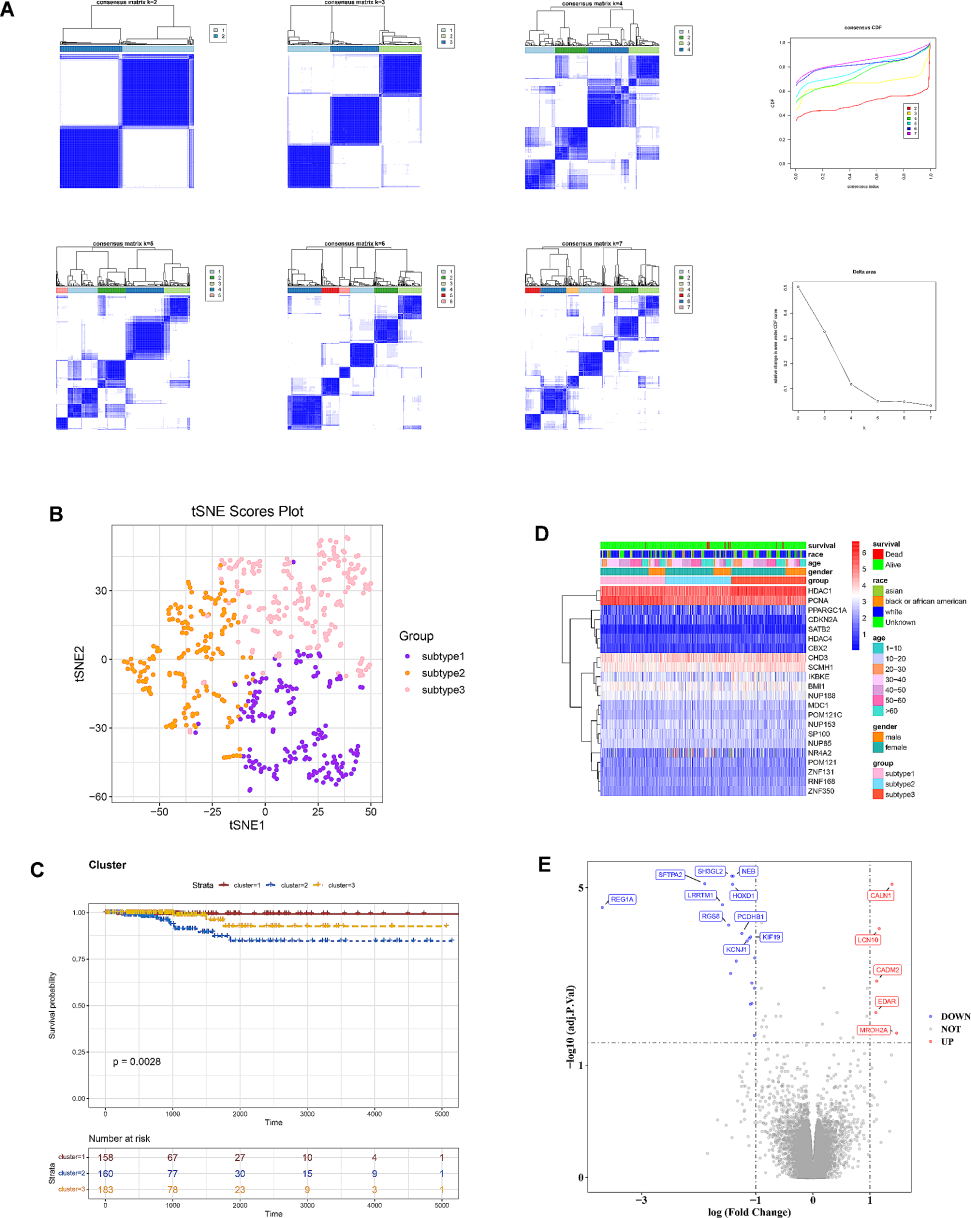
Identification of SUMOylation-related subtypes (**A**) Consensus clustering analysis of prognostic SRGs in PTC patients; (**B**) T-SNE analysis of 3 different subtypes; (**C**) Survival curves of 3 different subtypes; (**D**) Expression heatmap of prognostic-related SRGs in clinical characteristics of 3 different subtypes; (**E**) Volcano map of DEGs2 (Differentially expressed genes between two subtypes, cluster 1 and cluster 2)

The results of consistent clustering analysis suggested that the PTC samples in TCGA-PTC were clustered into three SUMOylation-related subtypes based on 22 prognosis-related SRGs (cluster 1, cluster 2 and cluster 3; Fig. 2A). The point map of the different subtypes showed that the 3 different subtypes could be better distinguished from each other (Fig. 2B). The results of the survival analysis suggested that cluster 1 and cluster 2 showed more significant survival differences among the three subtypes (Fig. 2C). The heatmap of the 22 prognosis-related SRGs expression in different subtypes of clinical features was shown in Fig. 2D.

By analyzing the differentially expressed genes of two subtypes, Cluster 1 and Cluster 2, we obtained DEGs2, which consists of a total of 24 genes with differential expression. Among these, 5 genes were found to be upregulated, while 19 genes were downregulated (Fig. 2E).

### Differential genes associated with SRGs were obtained

**Fig. 3:**
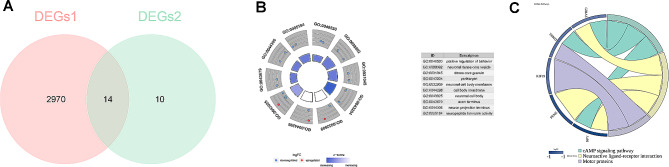
Identification and enrichment analysis of candidate genes (**A**) Venn diagram of DEGs1 (PTC vs. control) and DEGs2 (cluster 1 vs. cluster 2); (**B**) The top 10 GO pathways of candidate genes; (**C**) KEGG pathways of candidate genes

Totally 14 differential genes associated with SRGs (SFTPA2, HOXD1, TNNI3, LCN10, BMP8A, RGS8, IL1RAPL1, PENK, KIF19, GRIA3, SERPIND1, CADM2, NPY, and MROH2A) were identified by taking intersection of 2,984 DEGs1 (PTC vs. control) and 24 DEGs2 (cluster 1 vs. cluster 2), and the Venn diagram was shown in Fig. 3A.

Moreover, GO and KEGG pathway analyses of the 14 differential genes associated with SRGs were achieved to discover the potential biological functions. The GO results indicated that these genes were mainly involved in “neuronal cell body”, “positive regulation of behavior”, “receptor ligand activity” and etc. biological processes (Fig. 3B). Meanwhile, the KEGG results showed that the most significantly enriched pathways contained “cAMP signaling pathway”, “neuroactive ligand-receptor interaction”, “motor proteins”. (Figs. 3C).

### BMP8A, RGS8, and SERPIND1 were biomarkers

**Fig. 4:**
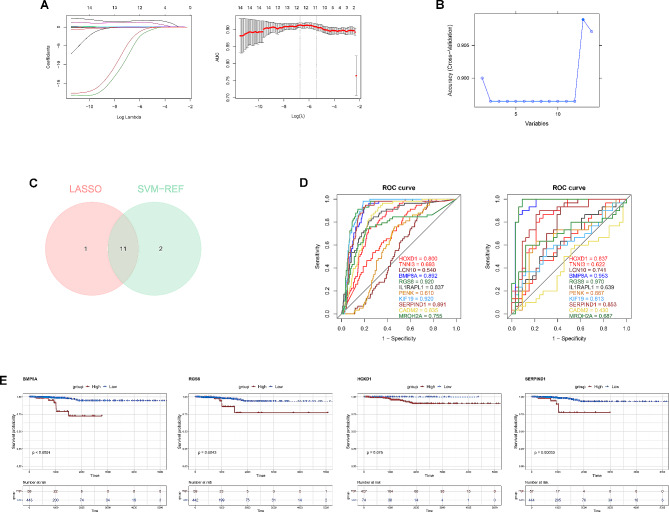
Screening of biomarkers (**A**) Screening of feature genes by LASSO regression; (**B**) Screening of feature genes by SVM-RFE; (**C**) Venn diagram of feature genes screened by LASSO and SVM-RFE; (**D**) ROC curves of candidate genes; (E) Survival curves for high and low expression group of candidate genes

A total of 14 differential genes associated with SRGs were submitted into LASSO, and the results showed that 12 feature genes (BMP8A, RGS8, HOXD1, TNNI3, LCN10, IL1RAPL1, PENK, GRIA3, SERPIND1, CADM2, MROH2A and KIF19) were obtained, as shown in Fig. 4A. The SVM-RFE method identified the top 13 genes with accuracy as feature genes (BMP8A, SERPIND1, RGS8, NPY, IL1RAPL1, CADM2, LCN10, HOXD1, PENK, KIF19, SFTPA2, MROH2A and TNNI3), as shown in Fig. 4B. Totally 11 candidate genes, BMP8A, RGS8, HOXD1, TNNI3, LCN10, IL1RAPL1, PENK, SERPIND1, CADM2, MROH2A and KIF19, were secured by intersecting the feature genes screened through machine learning algorithms (Fig. 4C).

Subsequently, the ROC curves of the candidate genes in the TCGA-PTC and GSE60542 datasets showed that the AUC values of BMP8A, RGS8, HOXD1, and SERPIND1 were all greater than 0.8 (Fig. 4D). Next, BMP8A, RGS8, and SERPIND1 were identified as biomarkers through the K-M survival curves, with the threshold of *P* < 0.05 (Fig. 4E).

### Pathways of biomarkers are acquired by GSEA

**Fig. 5:**
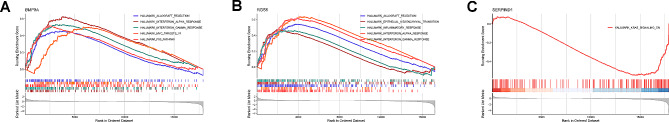
GSEA of biomarkers (**A**) GSEA of BMP8A; (**B**) GSEA of RGS8; (**C**) GSEA of SERPIND1

The results of GSEA suggested that both BMP8A and RGS8 were enriched in “allograft rejection”, “interferon alpha response” and “interferon gamma response” (Fig. 5A and B). SERPIND1 was enriched in “kras signaling dn” (Fig. 5C) (Supplementary Table S2).

### Biomarkers were associated with immune infiltrating cells

**Fig. 6:**
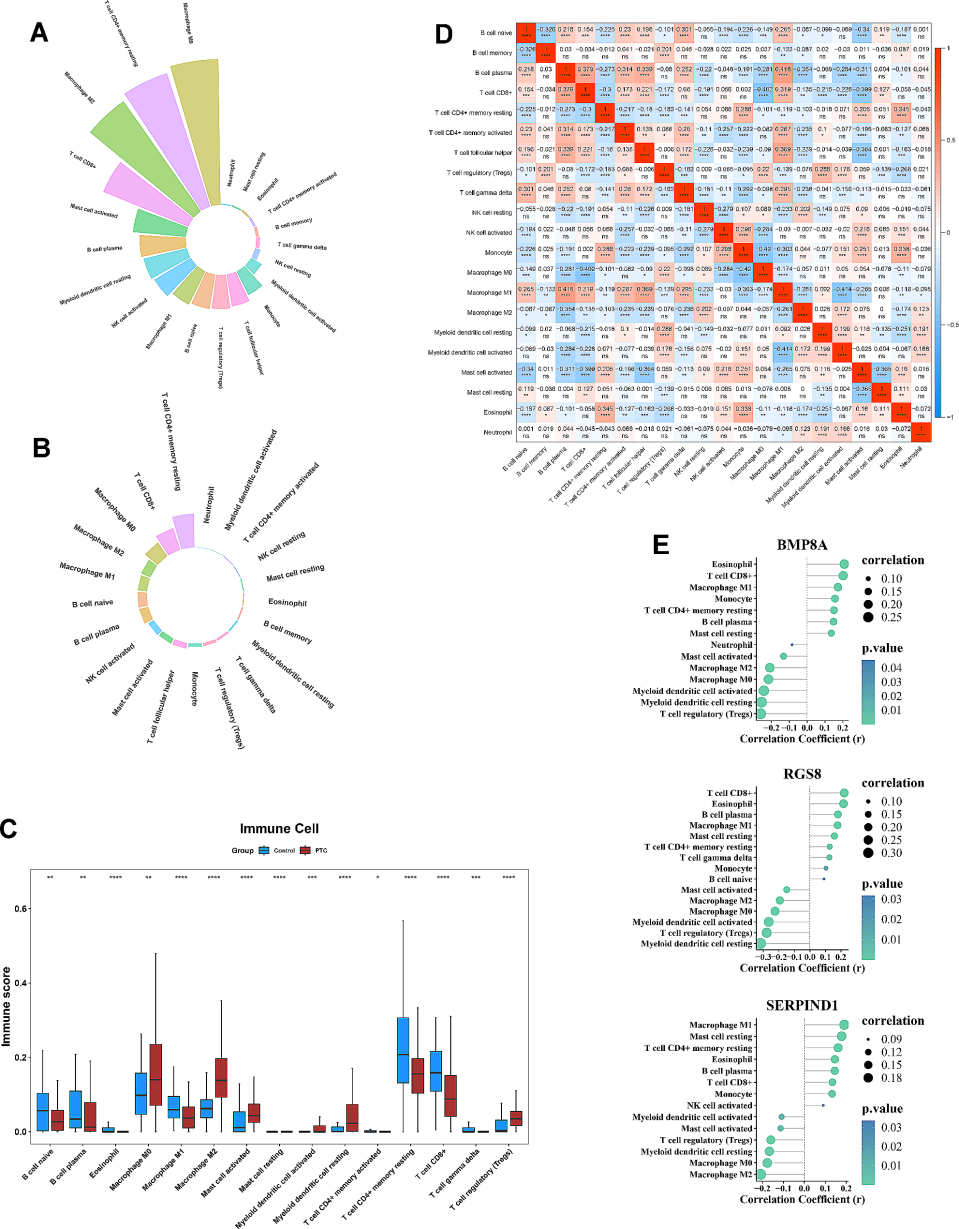
Immune cell infiltration analysis (**A**) Abundance of immune infiltrating cells for PTC samples in TCGA-PTC; (**B**) Abundance of immune infiltrating cells for control samples in TCGA-PTC; (**C**) Differential immune infiltrating cells between PTC and control samples in TCGA-PTC; (**D**) Correlation analysis between immune infiltrating cells; (**E**) Correlation analysis between biomarkers and differential immune cells

Assessment of the relative abundance of immune cells showed that abundances of M0 macrophage, resting memory CD4 + T cell and M2 macrophage were higher, abundances of neutrophil, resting mast cell and eosinophil were lower in PTC samples (Fig. 6A); while abundances of resting memory CD4 + T cell, CD8 + T cell and M0 macrophage were higher, and abundances of neutrophil, activated myeloid dendritic cell and activated memory CD4 + T cell were lower in control samples (Fig. 6B). The results of the immune score showed significant differences in 15 types of immune cells in PTC and control samples (Fig. 6C), including M1 macrophage, M2 macrophage, activated mast cell, etc. Correlation analysis among immune cells suggested that the highest positive correlation between M0 macrophage and monocyte (cor=-0.42), and the highest negative correlation between M1 macrophage and plasma B cell (cor = 0.416) were found (Fig. 6D). Meanwhile, correlation analysis between biomarkers and immune cells showed that BMP8A and RGS8 were positively correlated with eosinophil and CD8 + T cell, and negatively correlated with resting myeloid dendritic cell and regulatory T cell (Tregs); and SERPIND1 was positively correlated with M1 macrophage and resting mast cell, and negatively correlated with M0 macrophage and M2 macrophage (Fig. 6E).

### The IC50 of the drugs showed differences

**Fig. 7:**
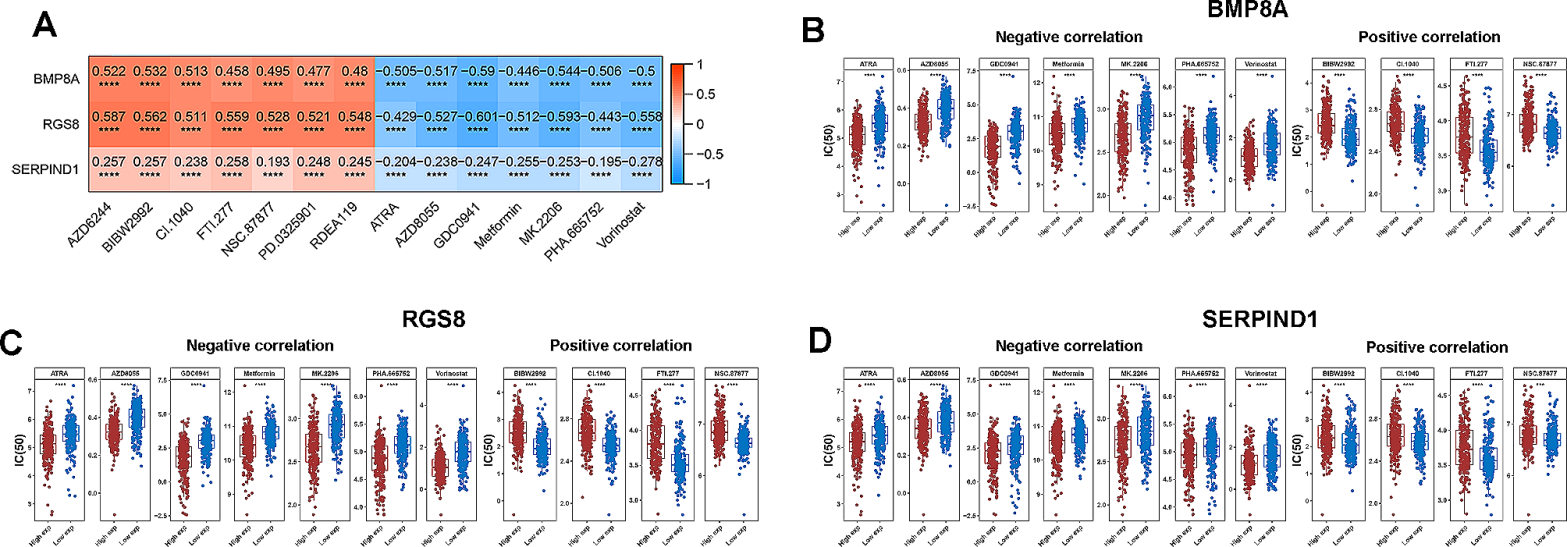
Drug sensitivity analysis (**A**) Heatmap of correlation between biomarkers and drug; (**B**) IC50 of the drugs in the high and low expression groups of BMP8A; (**C**) IC50 of the drugs in the high and low expression groups of RGS8; (**D**) IC50 of the drugs in the high and low expression groups of SERPIND1. (*p < 0.05; **p < 0.01; ***p < 0.001)

The correlation analysis between PTC biomarkers in the TCGA-PTC dataset and 138 drugs from the GDSC database revealed that GDC0941 exhibited the strongest correlation between BMP8A and RGS8 (Fig. 7A). Furthermore, drug sensitivity analysis results suggested that IC50s of the drugs in the high and low expression groups of each biomarker showed differences (Fig. 7B-D).

### BMP8A, RGS8, and SERPIND1 were down-regulated in PTC samples

**Fig. 8:**
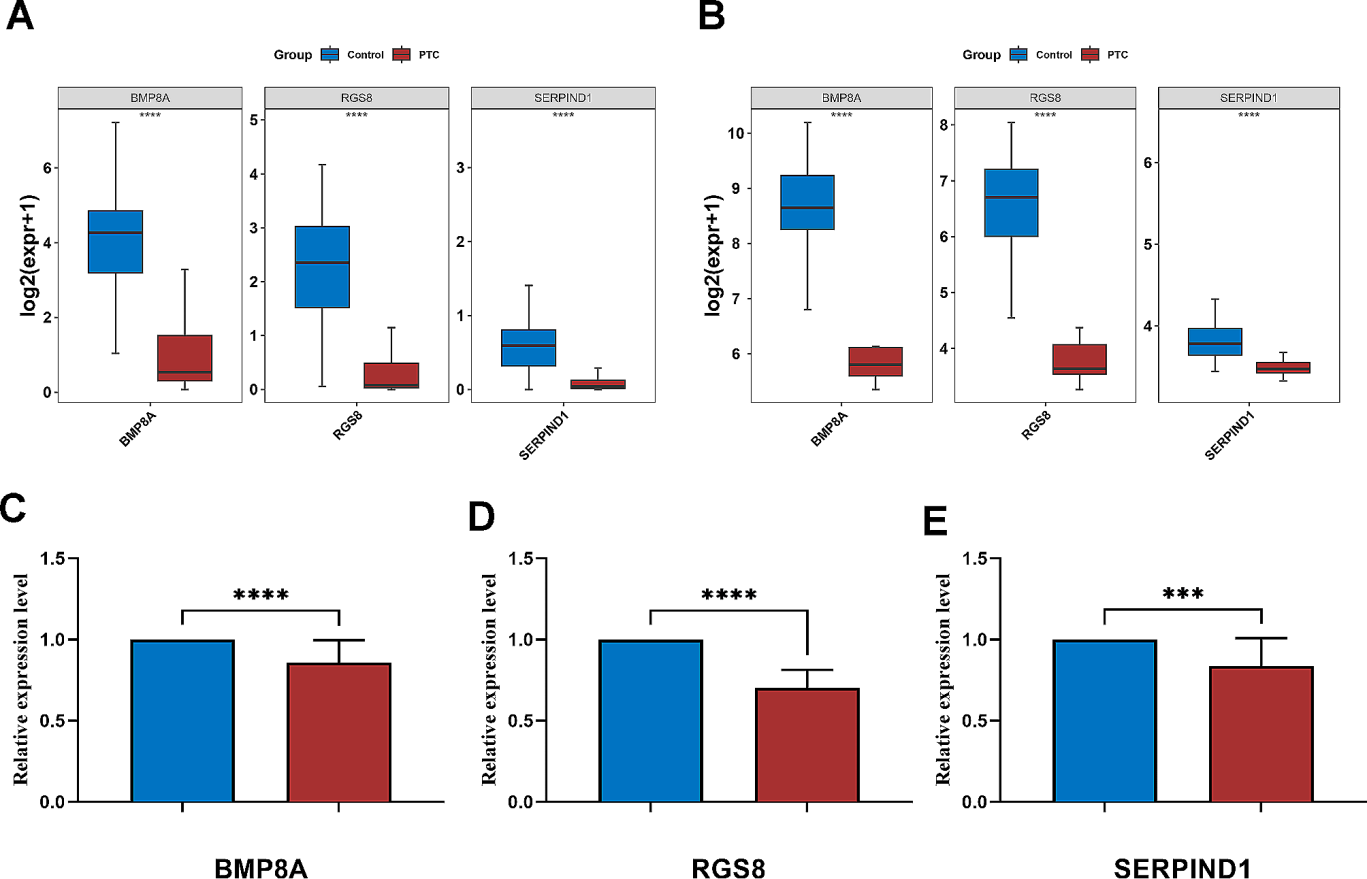
Expression validation of biomarkers (**A**) The expression level of biomarkers in the TCGA-PTC dataset; (**B**) The expression level of biomarkers in the GSE60542 dataset; (**C**) BMP8A expression in normal thyroid tissue and PTC; (**D**) RGS8 expression in normal thyroid tissue and PTC; (**E**) SERPIND1 expression in normal thyroid tissue and PTC. (*p < 0.05; **p < 0.01; ***p < 0.001)

The expression validation results of the biomarkers suggested that BMP8A, RGS8, and SERPIND1 were significantly down-regulated in PTC samples and showed consistent trends in TCGA-PTC and GSE60542 datasets (Fig. 8A-B).

Subsequently, we validated the expression of three prognostic genes in 20 PTC tissues and their adjacent normal tissues through Quantitative Real-time PCR (qRT-PCR) analysis. The results also indicate that the expression of these three prognostic genes is significantly downregulated in PTC tissue (Fig. 8C-E).

### Clinical data validation of the relationship between BMP8A expression and recurrence risk stratification

**Fig. 9:**
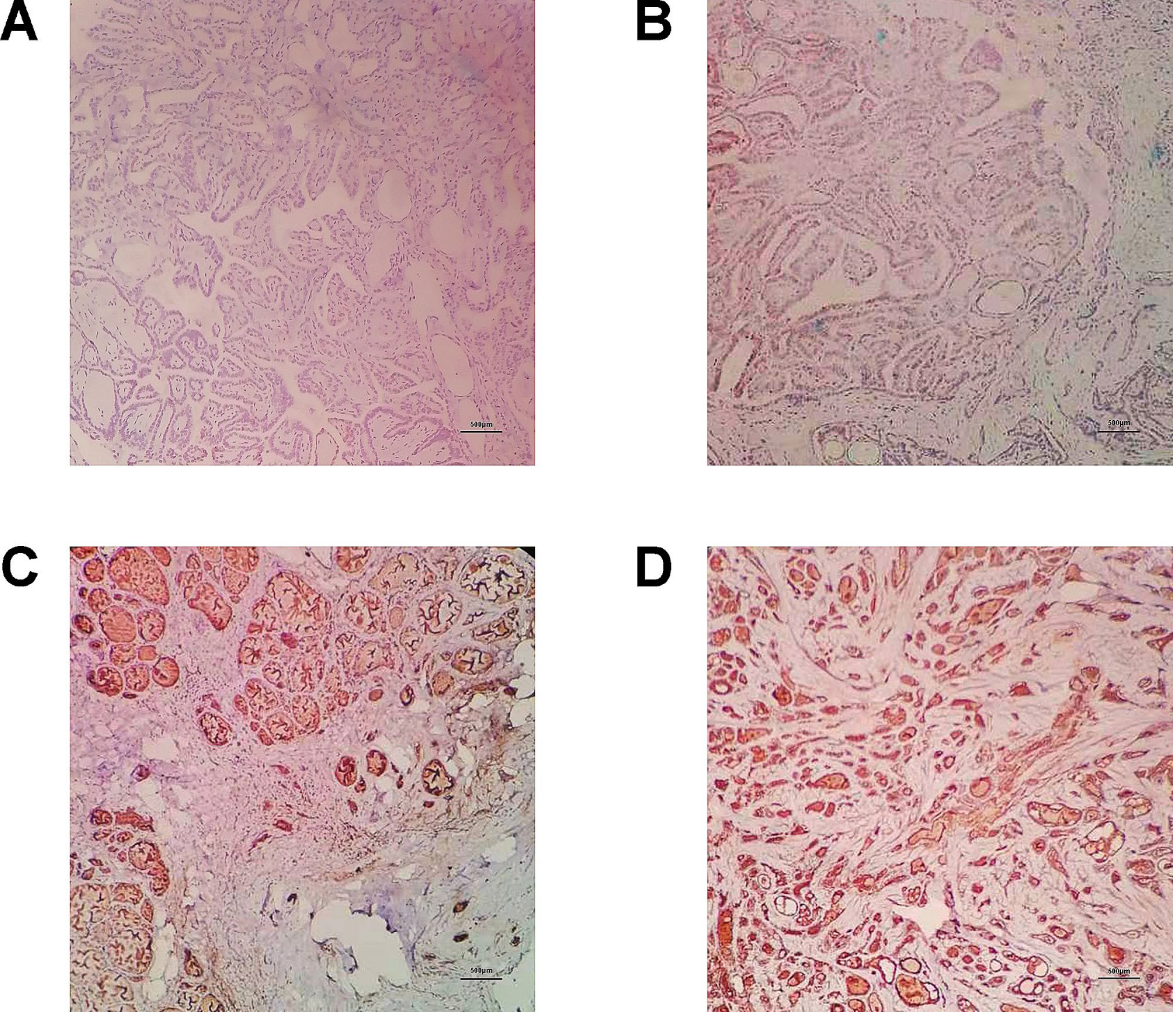
Immunohistochemical results of PTC patient specimens **A**-**B**. Negative expression of BMP8A in PTC tissues (IHC × 40); **C**-**D**. Positive expression of BMP8A in PTC tissues (IHC × 40)

We validated the relationship between BMP8A expression and recurrence risk stratification in patients with papillary thyroid carcinoma (PTC) by integrating other clinicopathological indicators. The expression of BMP8A in PTC samples was confirmed using immunohistochemistry (Fig. 9). Out of 120 PTC patient samples, 97 showed negative expression of BMP8A, while 21 were positive. Subsequently, we analyzed the relationship between BMP8A expression and patient recurrence risk stratification using corresponding clinicopathological data. The results indicated a strong negative correlation between BMP8A expression and recurrence risk stratification (*p* = 0.005) (Table 2). This finding is consistent with our analysis results from the TCGA database, confirming the role of BMP8A expression in predicting patient disease recurrence.

**Table 2:** Univariate and multivariate analysis of risk stratification for recurrence of papillary thyroid carcinoma

Covariates	Total	Univariate analysis		Multivariate analysis		
		x^2^	*p* value	Exp (B)	95% CI	*p* value
Gender		0.522	*p* = 0.47			
Male	32					
Female	88					
Age		0.61	*p* = 0.435			
<=55	91					
>55	29					
Pathologic T		14.445	*p* = 0.002			*p* = 0.054
T1	50			Reference		
T2	31			0.116	0.024–0.564	
T3	27			0.476	0.106–2.143	
T4	13			0.379	0.077–1.868	
Pathologic N		0.549	*p* = 0.018			*p* = 0.017
N0	68			Reference		
N1	52			0.216	0.062–0.756	
BMP8A		9.221	*p* = 0.002			*p* = 0.005
Negative	99			Reference		
Positive	21			0.147	0.038–0.567	

## Discussion

Papillary thyroid carcinoma (PTC) is generally characterized as a relatively indolent tumor, boasting long-term survival rates exceeding 95%. Nonetheless, certain aggressive forms of PTC lead to diminished disease-free and overall survival rates [26]. Notably, prevalent treatment approaches like total thyroidectomy and radioactive iodine therapy might result in overtreatment for many patients with a low-risk profile [27]. Consequently, there is an increasing emphasis on identifying novel biomarkers for PTC. A pertinent study highlighted that mRNA levels of SUMOylation-related proteins are significantly reduced in most PTC tissues compared to normal thyroid tissues [28]. Despite these initial findings, research specifically focusing on the role of SUMOylation-related biomarkers in PTC remains scant, underscoring the need for more targeted investigation in this area.

In our study, we identified three genes associated with SUMOylation modification—BMP8A, RGS8, and SERPIND1—as significant to the prognosis of papillary thyroid carcinoma (PTC) patients. Notably, patients with high expression of these genes exhibited a significantly reduced prognosis compared to those in the low-expression group. Further analysis confirmed that BMP8A, RGS8, and SERPIND1 are intricately involved in the initiation and progression of tumors, indicating their potential as prognostic markers in PTC [29–31].

BMP8A is part of the bone morphogenetic protein (BMP) gene family, which is known to encode secreted ligands of the transforming growth factor-β (TGF-β) protein superfamily. These ligands interact with various TGF-β receptors and activate SMAD family transcription factors, thereby influencing gene expression critical for a range of cellular functions from embryonic development and postnatal growth to tissue repair, regeneration, and organismal homeostasis [32]. The BMP signaling pathway is also implicated in inducing epithelial-mesenchymal transition, a key process in cancer progression [33]. Studies have established a strong correlation between BMP8A expression and prognosis in renal cell carcinoma and breast cancer, where high BMP8A expression typically signals a poorer prognosis [34, 35]. This effect may be due to BMP8A’s role in stimulating Nrf2 phosphorylation, thus affecting ROS balance and promoting tumor cell proliferation. In thyroid cancer research, Liu et al. [36] reported abnormal overexpression of BMP8A in thyroid cancer tissues. While our study corroborates the link between high BMP8A expression and poor prognosis in PTC patients, it interestingly notes a decreased expression of BMP8A in PTC tissues compared to normal thyroid tissues. Therefore, the precise relationship between BMP8A expression and PTC prognosis necessitates further investigation.

Genes encoding regulators of G-protein signaling (RGS) produce RGS proteins that are pivotal in modulating G-protein coupled receptors (GPCRs). The interaction between GPCRs and RGS proteins is well-established in the context of cancer onset and progression [37]. While various RGS family members have been implicated in malignant tumor development [38], information on RGS8 remains scarce. Current research has only identified its abnormal expression in brain and breast cancers [30], with the detailed mechanisms of its involvement in tumorigenesis still unclear. In the realm of thyroid cancer, Bai et al. [39] observed that RGS8 is typically downregulated in thyroid cancer tissues, a finding that aligns with the results of our study. Similar to BMP8A, there are no comprehensive studies yet that explore the relationship between RGS8 expression and the prognosis of papillary thyroid carcinoma, indicating an area ripe for further research.

Recent years have seen increasing research into the relationship between SERPIND1 and various malignant tumors. Specifically, SERPIND1 has been implicated in promoting the development of ovarian cancer by augmenting the phosphorylation of the PI3K/AKT pathway. This enhancement leads to increased proliferation, migration, invasion, and cell cycle regulation of ovarian cancer cells while concurrently inhibiting apoptosis [31]. Despite these insights, the association between SERPIND1 and thyroid cancer, particularly papillary thyroid carcinoma (PTC), remains unexplored, marking a significant gap in current thyroid cancer research.

To investigate the related pathways and biological functions of BMP8A, RGS8, and SERPIND1, we performed a comprehensive biological functional correlation analysis. The results from Gene Set Enrichment Analysis (GSEA) indicate that BMP8A and RGS8 share enrichment in several key pathways, including allograft rejection, interferon alpha response, and interferon gamma response. Conversely, SERPIND1 is predominantly associated with the kras signaling down pathway. Based on these findings, we postulate that these three genes may be intricately linked to the immune mechanisms contributing to tumor development and progression.

Immune cell infiltration is a pivotal component of the tumor microenvironment and significantly influences the initiation and progression of tumors [40]. Given the critical role of immune factors in the progression of papillary thyroid carcinoma (PTC) [41, 42], our study aimed to elucidate the correlations between BMP8A, RGS8, and SERPIND1 and immune cell infiltration. We discovered positive correlations of these genes with immune cells known for their anti-tumor properties, such as eosinophils, CD8 + T cells, M1 macrophages, and resting mast cells. Conversely, negative correlations were observed with immune cells typically associated with tumor promotion, including resting myeloid dendritic cells, regulatory T cells (Tregs), M0 macrophages, and M2 macrophages. Drawing on existing knowledge about immune cells’ roles in PTC, it is evident that eosinophils, CD8 + T cells, and M1 macrophages generally exhibit anti-tumor effects, whereas Tregs, M0 macrophages, and M2 macrophages contribute to tumor development [43]. Therefore, it can be inferred that downregulation of BMP8A, RGS8, and SERPIND1 might lead to a shift in the immune microenvironment towards a pro-tumor state, thus facilitating the occurrence and advancement of PTC.

To comprehensively assess the relationship between identified biomarkers and responses to chemotherapy and molecular targeted therapy, we determined the half-maximal inhibitory concentration (IC50) values of widely used drugs in relation to BMP8A, RGS8, and SERPIND1. Our comparative analysis identified a pronounced correlation between GDC0941, also known as Pictilisib, and the expression levels of BMP8A and RGS8. Notably, higher expression of these genes correlated with significantly improved efficacy of GDC0941. Pictilisib, a PI3K inhibitor, is instrumental in regulating key cellular functions including proliferation, differentiation, and apoptosis by suppressing PI3K activation and thus, inhibiting tumor cell proliferation and inducing apoptosis [44–46]. While it is predominantly used in treating breast cancer [47, 48], emerging evidence suggests its broader application across various malignancies, including thyroid cancer [49–51]. Notably, studies by Burrows et al. [52, 53] and Kandil et al. [54] have demonstrated Pictilisib’s efficacy in inhibiting metastasis and reducing the growth of thyroid cancer cells, respectively. These insights underscore the potential of targeting the PI3K/AKT signaling pathway in thyroid cancer treatment, offering promising avenues for future therapeutic strategies.

Subsequently, we selected BMP8A, the gene with the highest expression among the three we identified, to validate its role in predicting the prognosis of PTC patients. Since the mortality rate of PTC patients is quite low, our primary evaluation focused on the relationship between BMP8A expression and tumor recurrence in PTC patients. Currently, the risk of recurrence in PTC patients is clinically assessed based on the ATA guidelines, which categorize patients into low, intermediate, and high-risk groups. Due to the increased public awareness of health and advancements in medical imaging technology, most patients are diagnosed and treated for PTC at an early stage. Consequently, there are few high-risk patients in our center; therefore, we divided the patients into low-risk and intermediate-high-risk groups [55, 56]. We discovered that the expression of BMP8A in the tumor tissues of patients in the intermediate-high-risk group was significantly higher than that in the low-risk group. Hence, BMP8A may serve as a valuable supplement in predicting the recurrence risk of PTC patients.

This study successfully identified SUMOylation-related biomarkers in papillary thyroid carcinoma (PTC) using a series of bioinformatics approaches. It delved into their relationships with tumor subtypes, clinical features, and the immune microenvironment, laying the groundwork for new theoretical approaches in PTC research and treatment.

In subsequent clinical practice, we can initially conduct BMP8A testing on PTC patients and tailor postoperative management accordingly based on the test results. For instance, for BMP8A-positive patients, we can consider shortening their follow-up intervals, implementing more effective TSH inhibition treatment, and assessing outcomes through long-term follow-up.

However, this study is not without limitations. While the focus was specifically on PTC, other types of thyroid cancer warrant similar in-depth exploration to understand the broader implications of SUMOylation in thyroid malignancies. In this study, we only validated the association between BMP8A expression and prognosis in clinical patients, however, further validation is required for the other two genes.

## Conclusions

Conclusively, SUMOylation-related biomarkers, particularly BMP8A, RGS8, and SERPIND1, demonstrate promising prognostic value, offering insights into the immune microenvironment of PTC and potential therapeutic pathways. These markers show great potential in diagnosing and predicting the prognosis of PTC patients.

### Electronic supplementary material

Below is the link to the electronic supplementary material.

Supplementary Material 1

Supplementary Material 2

## Data Availability

No datasets were generated or analysed during the current study.

## Abbreviations

BMP: Bone morphogenetic protein
DEGs: Differentially expressed genes
GEO: Gene Expression Omnibus
GO: Gene Ontology
GPCRs: G-protein coupled receptors
GSEA: Gene set enrichment analysis
IC50: 50% inhibitory concentration
K-M: Kaplan-Meier
KEGG: Kyoto Encyclopedia of Genes and Genomes
LASSO: Least absolute shrinkage and selection operator
MSigDB: Molecular Signatures Database
PTC: Papillary thyroid carcinoma
RGS: G-protein signaling
ROC: Receiver operating characteristic
SRGs: Small ubiquitin-like modifier modification -related genes
SUMOylation: Small ubiquitin-like modifier modification
t-SNE: T-distributed stochastic neighbor embedding
TCGA: The Cancer Genome Atlas
THCA: Thyroid cancer
Tregs: Regulatory T cell
TSH: Thyroid stimulating hormone
